# Off-stoichiometry improves the photostructuring of thiol–enes through diffusion-induced monomer depletion

**DOI:** 10.1038/micronano.2015.43

**Published:** 2016-02-15

**Authors:** Mikael Hillmering, Gaspard Pardon, Alexander Vastesson, Omkar Supekar, Carl Fredrik Carlborg, Birgit D. Brandner, Wouter van der Wijngaart, Tommy Haraldsson

**Affiliations:** 1 Micro and Nanosystems, KTH Royal Institute of Technology, Osquldas väg 10, SE-10044, Stockholm, Sweden; 2 SP Chemistry, Materials and Surfaces, SP Technical Research Institute of Sweden, Drottning Kristinas väg 45, SE-114 28, Stockholm, Sweden

## Abstract

Thiol–enes are a group of alternating copolymers with highly ordered networks and are used in a wide range of applications. Here, “click” chemistry photostructuring in off-stoichiometric thiol–enes is shown to induce microscale polymeric compositional gradients due to species diffusion between non-illuminated and illuminated regions, creating two narrow zones with distinct compositions on either side of the photomask feature boundary: a densely cross-linked zone in the illuminated region and a zone with an unpolymerized highly off-stoichiometric monomer composition in the non-illuminated region. Using confocal Raman microscopy, it is here explained how species diffusion causes such intricate compositional gradients in the polymer and how off-stoichiometry results in improved image transfer accuracy in thiol–ene photostructuring. Furthermore, increasing the functional group off-stoichiometry and decreasing the photomask feature size is shown to amplify the induced gradients, which potentially leads to a new methodology for microstructuring.

## Introduction

Thiol–enes are a group of alternating copolymers with highly ordered networks and are widely used in applications ranging from high-performance coatings to optical, biomedical, sensing, and bioorganic modification applications^1–6^. A thiol–ene network is typically formed via radical-mediated step polymerization. Thiol and ene groups react in a 1:1 fashion through a so-called “click” reaction, with high yield and minimal side reactions when electron-rich enes are used. A chain transfer step, whereby a carbon-centred ene radical transfers to the thiol, determines the network formation rate and leads to delayed gelation, which results in very low shrinkage stress. These properties are widely exploited in materials technologies, in information and communications technologies, and in life sciences applications^4,7–10^.

Stoichiometric formulations, having a matching number of thiol and ene groups, enable full conversion of reactive groups into a highly cross-linked copolymer. However, intentional deviation from stoichiometric thiol–ene formulations has recently garnered interest^11–14^. Such off-stoichiometric formulations enable the creation of polymers containing a predictable amount of residual reactive groups, in which a thiol or ene excess in the prepolymer formulation remains in the final polymer. The residual active groups enable the tuning of mechanical properties and the development of improved surface modification processes while retaining most of the benefits offered by the underlying thiol–ene click chemistry^2,11–13^. Enhanced sensitivity for holographic data storage in off-stoichiometric formulations of thiol–enes has also been predicted^14^.

The thiol–ene reaction is commonly photoinitiated, which enables photostructuring of the polymer^15,16^. However, photostructuring of stoichiometric thiol–ene formulations requires high radical inhibitor concentrations to prevent severe feature broadening^10^. By contrast, we recently observed that highly off-stoichiometric thiol–ene (OSTE) formulations seem to allow for photostructuring with reduced structure broadening^17^, although this improvement was not quantified and the underlying mechanisms were not understood.

Here, we investigate the role of off-stoichiometry in the photostructuring of thiol–enes using confocal Raman and bright-field microscopy. We report on the discovery of significant differences between stoichiometric and OSTE formulations in terms of spatial distribution of chemical groups in zones immediately adjacent to the interface between the illuminated and non-illuminated regions (this interface is hereafter called the “photomask feature boundary,” PFB) and in terms of photostructuring quality. We also observe that diffusion of species during photostructuring induces depletion of the deficient enes in the case of an off-stoichiometric formulation with thiol excess. Finally, we propose an explanation for the underlying mechanism and investigate the effect of various parameters during photostructuring, such as light intensity, temperature, and mask effects.

Our findings reveal a new mechanism underlying photostructuring, which is particularly interesting for the development of thiol–ene polymers and their applications.

## Materials and methods

### Chemicals

The monomers used in this work were pentaerythritol tetrakis (2-mercaptoacetate) (PETMA) (Mercene Labs AB, Stockholm, Sweden) as the thiol monomer and 1,3,5-triallyl-1,3,5-triazine-2,4,6 (1H,3H,5H)-trione (TATATO) (Mercene Labs AB) as the ene-functional allyl-monomer. The photoinitiator was Lucirin TPO-L (Mercene Labs AB). The activity of the initiator batches is influenced by storage conditions; hence, experimental testing of the TPO-L amount was necessary prior to usage. All chemicals were used as received.

### Sample preparation and photostructuring conditions

Stoichiometric prepolymer mixtures were prepared by mixing 56.55 %w/w PETMA and 43.45 %w/w TATATO. Off-stoichiometric mixtures were prepared so that they had 80% thiol group excess (unless otherwise stated) by mixing 70.08 %w/w PETMA and 29.92 %w/w TATATO. In both mixtures, 1 %w/w TPO-L was added (unless otherwise stated). Mixing was performed using a vortexer for 2 min, followed by degassing using a desiccator for 20 min at −0.4 bar (unless otherwise stated). The prepolymer mixtures were always used fresh, i.e., within 1 hour before photostructuring and were kept in the dark until usage to prevent the formation of oligomers in the prepolymer prior to photostructuring. All samples (unless otherwise stated) consisted of 130-µm-thick UV-cured polymer films on combined glass–silicon–polymer substrates (for more information regarding how samples were manufactured, see Supplementary Figure S1). To minimize light scattering from the glass substrate during exposure, a rectangular piece cut from a silicon wafer was adhered onto a microscope glass slide using double-sided tape. To avoid disturbance from the silicon during Raman microscopy, a 500-µm-thick layer of the same polymer was polymerized onto the silicon. Finally, the prepolymer was UV-cured in a sandwich between the glass–silicon–polymer substrate and a 130-µm-thick cover glass. The layer thickness of the prepolymer was 130 µm, which was determined by using 130-µm-thick glass spacers placed between the substrate and the glass cover.

Sample polymerization was triggered by illumination with a collimated (3° collimation half-angle) near-UV short-arc mercury lamp at 12 mW cm^−2^ (unless otherwise stated) purchased from OAI Milpitas, San Jose, CA, USA.

During the photostructuring process, a photomask was placed on the top glass cover. Photomasks were of high-quality, transparent, and purchased from Micro Lithography Services Ltd, South Woodham Ferrers, UK (unless otherwise stated). Exposure times during photostructuring were 2 s and 3.5 s for the stoichiometric and off-stoichiometric mixtures, respectively (unless otherwise stated). The exposure times for the two mixtures were optimized experimentally to obtain complete curing while avoiding overexposure due to scattered light in the non-illuminated zone. Because network formation occurs more rapidly in a stoichiometric mixture, the required exposure dose was smaller. All samples (unless otherwise stated) were subsequently investigated using confocal Raman microscopy within 60 min after photostructuring to minimize spontaneous gelation.

### Photostructuring of stoichiometric and off-stoichiometric formulations

To investigate the effect of stoichiometry on the photostructuring of thiol–enes, stoichiometric and off-stoichiometric samples were prepared (as described in the “Sample preparation and photostructuring conditions” section) and photostructured using a photomask with patterns consisting of several dark circular areas 50 μm in diameter.

To investigate the dependence of deficient monomer depletion on the structure volume, defined by the photomask feature dimensions and the sample thickness, an off-stoichiometry thiol–ene sample was prepared and photostructured using a photomask with dark circular features 30, 60, 125, 250, 500 and 1000 μm in diameter (see Supplementary Figure S2). All features were exposed simultaneously through the photomask to ensure an identical polymerization history for all hole sizes.

### Photostructuring quality

Photostructuring quality was investigated by measuring the spatial shift, or broadening, of the transferred PFB image produced via photostructuring of thiol–enes at different stoichiometries and at different UV-light exposure times. This was performed using bright-field microscopy and digital image measurements (see Supplementary Figure S3 and S4 and Table S1 for experimental details). Sample mixtures with different stoichiometries of 0, 20, 40, 60, and 80% thiol group excess were prepared. For these samples, a photomask with straight pattern features was used during structuring. The photostructuring was performed using the same procedure as described in the section “Sample preparation and photostructuring conditions”, but the bottom glass substrate and the bottom polymer layer were replaced by a silicon substrate to reduce light reflection at the different interfaces. The broadening measurement was performed using a systematic measurement protocol of microscopy images. Measurements were performed on six samples per stoichiometry ratio for each of two sets of experiments, i.e., using different prepolymer batches and measuring on different days.

### Influence of temperature, light intensity, and mask effects

To verify that diffusion is the key mechanism, an experiment was designed to enable the observation of varying diffusivities, reaction rates, and mask effects during photostructuring. Three samples were prepared: an off-stoichiometric sample that was photostructured at 11 °C (cooled with a Peltier element) and exposed for 90 s at 10 mW cm^−2^; a second off-stoichiometric sample that was photostructured at 24 °C and exposed for 65 s at 10 mW cm^−2^; and a third stoichiometric sample that was photostructured at 24 °C and exposed for 35 s at 10 mW cm^−2^. All samples were mixed as described in the section “Sample preparation and photostructuring conditions”, except that the TPO-L photoinitiator concentration was 0.05 %w/w to reduce the light intensity attenuation with increasing depth in the sample and to allow for the observation of potential mask effects using confocal Raman microscopy depth scans. The mixing was performed using a centrifugal mixer at 2 × 15 s at 1500 rpm (model DAC 150.1 FVZ SpeedMixer™; Synergy Devices Ltd., High Wycombe, United Kingdom). The illumination of each sample resulted in a spatially varying light intensity, which was directly due to the 3° half-angle collimation of the light source. Instead of a plastic foil mask, here a chromium-on-glass photomask was used to achieve a sharp PFB. The non-illuminated pool region was wide, >1 mm, resulting in an approximately infinite pool of un-polymerized monomers. After exposure, each sample was investigated using confocal Raman microscopy (as described in the section Confocal Raman microscopy measurements), performing a vertical yz-scan across the PFB (see Supplementary Figure S5).

### Confocal Raman microscopy measurements

Sample characterization was performed using optical and confocal Raman microscopy (combined confocal Raman microscope/AFM/SNOM alpha300 RAS; WITec, Ulm, Germany) at an excitation wavelength of 532 nm. All measurements were performed at room temperature. The Raman spectra of both pure monomers and uncured formulations were first measured using point measurements to identify thiol and allyl peaks and to create a sum filter for the intensity from only thiol and allyl peaks (for more information, see Supplementary Figure S6). The thiol peak was identified at ~2575 cm^−1^, and the thiol Raman scattering signal was integrated between 2507 cm^−1^ and 2647 cm^−1^. The allyl peak was identified at ~1650 cm^−1^, and the allyl Raman scattering signal was integrated between 1627 cm^−1^ and 1667 cm^−1^. Furthermore, the carbon–oxygen double-bond peak was identified at ~1750 cm^−1^. Because inert C=O chemical bonds are present in both the thiol and ene monomers, this peak was used for normalization of the spectra (for more information, see Supplementary Figure S7). Thereafter, the spatial distribution of unreacted groups of PETMA and TATATO were measured using the Raman confocal microscopy of UV photostructured stoichiometric and off-stoichiometric thiol–ene samples. During the measurements, a 60 × magnification objective was used. Finally, the measured intensities were scaled to functional group molarity to obtain quantitative data. For this, a linear regression was performed on the results obtained for the pure monomers and uncured formulations (for more information, see Supplementary Figure S7).

## Results

### Off-stoichiometry causes the formation of a microscopic densely cross-linked rim at the PFB

**Figure 1:**
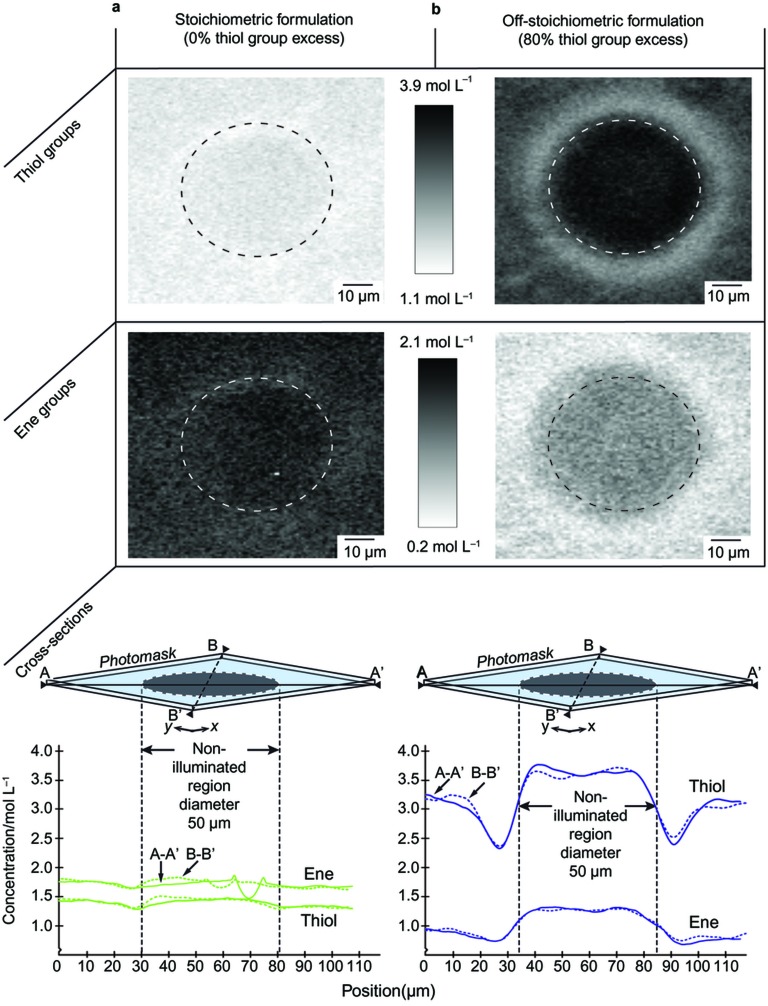
Photostructured (**a**) stoichiometric and (**b**) off-stoichiometric thiol–ene copolymer formulations imaged using confocal Raman microscopy. Two cross-sections (as indicated by diagonal dashed black lines on the photomasks in the bottom figures: A-Aʹ and B-Bʹ) were recorded from each image, showing a colour map of thiol group (top row) or ene group (second row) concentration. The circular dashed lines in each image correspond to the position of the non-illuminated region under the photomask, which was a 50-µm-diameter circular pattern. Cross-sectional concentration plots are shown below the images.

Photostructuring of a stoichiometric thiol–ene prepolymer formulation (Figure 1a) results in a low and almost flat concentration profile across the PFB for both thiol and ene groups. This indicates a high polymerization conversion rate in both the illuminated and the non-illuminated regions. By contrast, photostructuring of the OSTE formulation (Figure 1b) revealed distinctly varying concentration profiles of thiol and ene groups across the photomask edge. In the non-illuminated region, a high concentration of unreacted groups indicates no or limited polymerization. In the illuminated region, an intermediate concentration of unreacted thiol groups indicates incomplete conversion as controlled by the off-stoichiometric formulation. Most interestingly, immediately adjacent to the non-illuminated masked circular regions, a low concentration of unreacted groups, visible as bright ‘rims’ in the Raman images, indicates a high degree of polymerization with few residual reactive groups. In this narrow rim, a higher total conversion of monomers is achieved compared with that in the surrounding bulk polymer, which results in a locally denser polymer network.

### Off-stoichiometry reduces photostructure broadening

**Figure 2:**
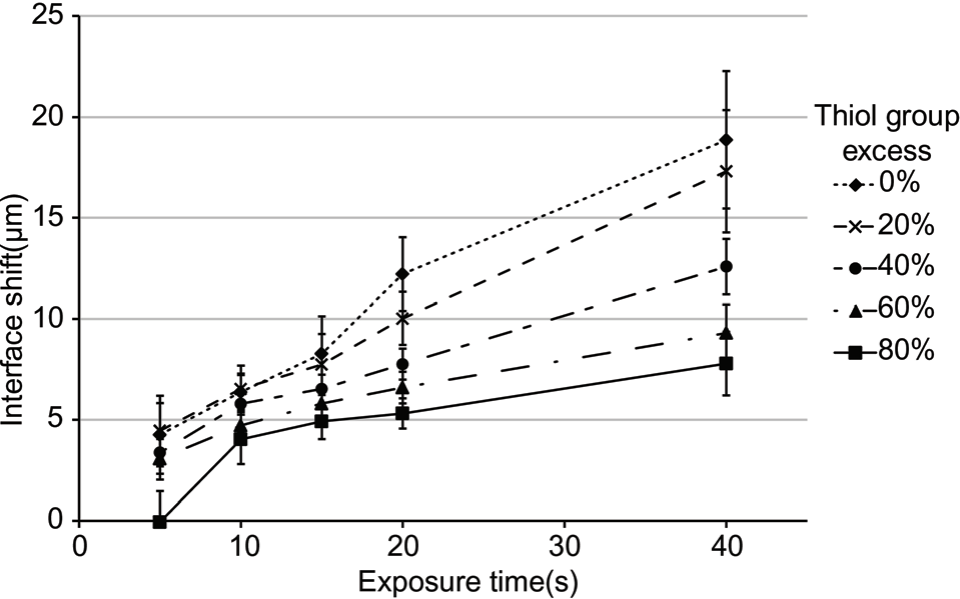
Measurements of the spatial shift in the transferred images in the polymer of a straight photomask edge for different exposure times and formulation stoichiometric ratios (*n* = 12). The graph demonstrates reduced broadening for highly off-stoichiometry formulations.

By varying the off-stoichiometric ratio of thiol:ene (i.e., the percentage of thiol group excess), a reduced structure broadening was observed (Figure 2). Compared with an on-stoichiometric ratio, an 80% thiol group excess resulted in significantly less broadening, even if the samples were overexposed for >20 s (*n* = 12). Thus, this result suggests that increasing the off-stoichiometry strongly reduces feature broadening, independently of exposure time.

### Diffusion-induced deficient ene group depletion

**Figure 3:**
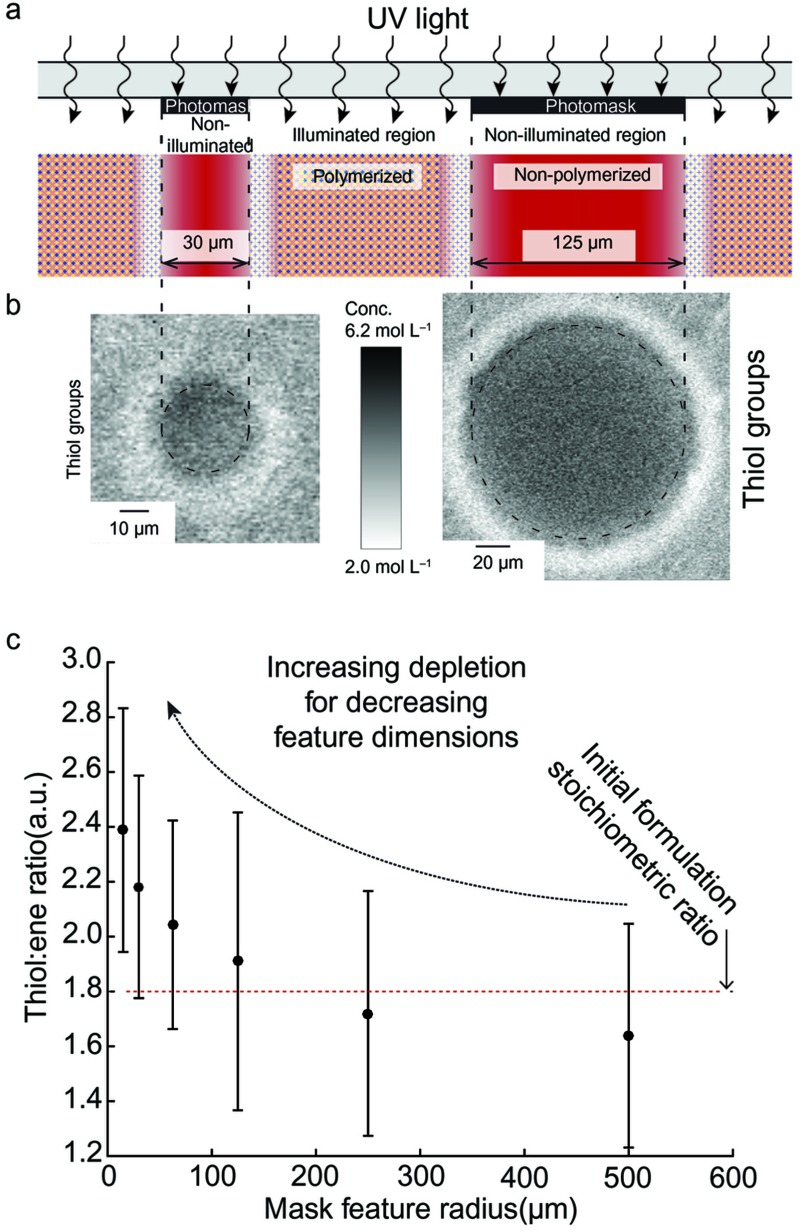
Decreasing the structure dimensions leads to an increasing depletion of the deficient ene groups in the non-illuminated region in 80% OSTE. (**a**) Schematic cross-section of the photostructuring. (**b**) The corresponding confocal Raman microscopy images of unreacted thiol groups in large-diameter (125 µm) and small-diameter (30 µm) features. (**c**) Measured ratio of unreacted thiols over enes as a function of the feature radius.

When the diameter of the non-illuminated area of the photomask was decreased (Figure 3a and b), an increase in the thiol:ene concentration ratio was observed in the non-illuminated region (Figure 3c). This result suggests that a greater relative proportion of the deficient allyl monomer ene groups is being depleted through diffusion into the illuminated region as the volume under the non-illuminated area shrinks.

## Discussion

**Figure 4:**
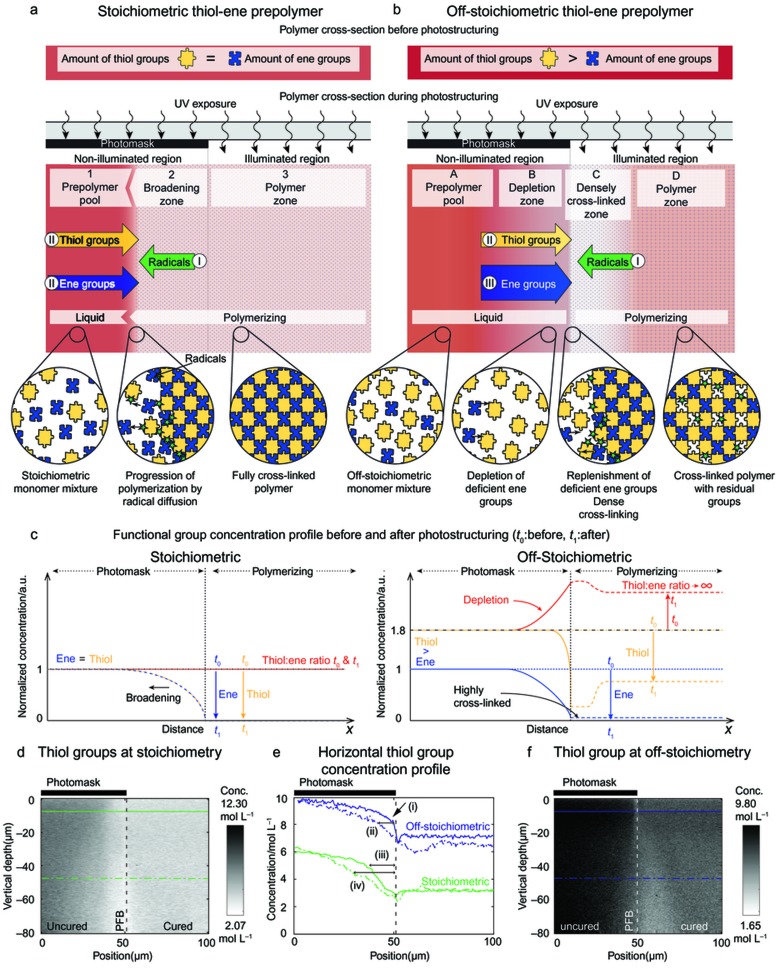
Schematic description of the network formation at a photomask edge in thiol–enes. (**a**) Stoichiometric formulation case: diffusion of radicals (I) from zone 3 to zone 2 during exposure leads to broadening of the photostructuring (= zones 2 and 3) due to reactions with the available ene and thiol functional groups (II). (**b**) Off-stoichiometric formulation case: radical diffusion (I) occurs, but in this case, the counter-acting diffusion of thiol (II) and, importantly, ene monomers (III) from the non-illuminated region to the illuminated region leads to the formation of two distinct zones: zone C with a local denser cross-linking and conversion, and zone B with a depletion of the deficient groups. (**c**) Schematic plot of the functional group concentrations and their ratio before and after photostructuring (**d**) and (**f**): Vertical Raman cross-section of thiol groups present in stoichiometric (**d**) and off-stoichiometric (**f**) thiol–ene after photostructuring. (**e**) Cross-section plot recording along the solid and dashed horizontal lines (**d**) and (**f**). Stoichiometric and off-stoichiometric concentration profiles show that light spreading causes the following features: in the off-stoichiometric case, a sharp interface (i) is achieved, and broadening under the photomask (ii) is diminished compared with the stoichiometric case, where broadening is already evident on the 5-µm-deep cross-section (iii) and significantly wider at 50 µm of depth (iv).

To understand the observed phenomena, we need to first consider the different processes occurring during the photostructuring of stoichiometric and off-stoichiometric formulations (Figure 4).

During photostructuring of a photoinitiated prepolymer formulation, cross-linking in the illuminated region causes species concentration differences between the illuminated and non-illuminated regions, inducing molecular diffusion. Radicals are created in the illuminated regions and diffuse into the non-illuminated regions (I). In the illuminated region, the forming network rapidly incorporates any available monomers, causing monomer diffusion from the non-illuminated pool of liquid prepolymer into the illuminated region (II).

In a stoichiometric formulation, this gives rise to three distinct zones (Figure 4a). In the non-illuminated region, there exists (1) a prepolymer pool, which is a zone consisting of unpolymerized bulk prepolymer, and forms (2) a broadening zone, which is the zone immediately adjacent to the illuminated zone, where diffusion of radicals from the illuminated region into the non-illuminated region triggers polymerization, and hence structure broadening. In the illuminated region (3), a polymerizing zone forms, where rapid conversion occurs due to the continuous production of radicals in the presence of a stoichiometric ratio of reactive groups.

In an off-stoichiometric formulation, because of the intentional imbalance in the concentrations of reactive groups, molecular diffusion dramatically affects the polymerization conditions in the vicinity of the PFB (Figure 4b). Based on the findings reported in Figure 1, four zones are formed. In the non-illuminated region, there is (A) a prepolymer pool, which is a zone consisting of unpolymerized prepolymer, and forms B) a depletion zone, which is a non-polymerizing and widening zone immediately adjacent to the PFB characterized by the depletion of the deficient ene groups. In the illuminated region, a densely cross-linked zone (C) forms a polymerizing zone, which is immediately adjacent to zone B, where rapid and higher conversion occurs, and (D) an off-stoichiometric polymer zone, which is a polymerizing zone where slower and incomplete conversion occurs as dictated by the off-stoichiometric formulation.

The formation of zones B and C occurs via a clearly different mechanism compared with that in stoichiometric formulations. Because of the intentional off-stoichiometry mixing ratio, polymerization in illuminated regions, i.e., zones C and D, causes a rapid consumption of the deficient groups. By contrast, in zone C, the deficient groups are continuously replenished by the diffusion of thiol (II) and, importantly, ene (III) monomers from the non-illuminated region. The larger replenishment of the deficient ene groups comparatively increases the conversion rate in zone C compared with zone D, as the step-growth polymerization is a second-order reaction with respect to the thiol and ene group concentrations. This stoichiometric imbalance rapidly causes a diffusion barrier in the form of a densely cross-linked polymer “rim” in zone C, while the deficient groups become strongly depleted in zone B. Consequently, cross-linking is hindered in zone B because of depletion of the deficient groups and because of the strongly reduced diffusion of initiating radicals from the illuminated region into the non-illuminated region (I) due to the rapid wall formation in zone C.

Until the wall formation occurs, oligomers forming in zone C diffuse into zone B, although at a low rate because of high molecular weight^18^. This reaction is expected to produce two counteracting effects: it contributes to an increase in the partial conversion in zone B while it decreases the polymerization rate in zone B, as the oligomers mostly contain unreacted excess thiol groups. As seen in Figure 4d (and Supplementary Figure S5), during photostructuring of the stoichiometric sample, it is evident that incident UV-light spreading causes functional group conversion under the photomask. This effect causes structure broadening, which increases with depth into the non-illuminated region adjacent to the PFB (Figure 4d). This undesired polymerization in the non-illuminated region correlates with previously observed feature broadening^10,17^. As seen in the off-stoichiometric case (Figure 4f), broadening under the photomask is diminished because radical diffusion is rapidly hindered by the densely cross-linked ‘rim’ forming in the illuminated area adjacent to the PFB coupled with the simultaneous depletion of ene groups close to the forming structure. Furthermore, the cross-linked ‘rim’ widens with increasing depth in the illuminated area. This widening is due to the reduced reaction rate and hence increased diffusion length (of ene groups) resulting from the light spreading and light attenuation under the mask edge shadow and from the correlated slower viscosity build-up in the illuminated area adjacent to the mask edge compared with higher up in the sample. The role of diffusion is diminished at low temperatures (~11 °C), and the resulting cross-linked illumination interface around the PFB is diffuse (see Supplementary Figure S5). This effect is due to the combination of very low diffusion of the functional groups in the very viscous sample and the spreading of light at the PFB.

Consequently, the following outcomes are expected. First, the enrichment–depletion effect (II) in zone C and B is triggered by the off-stoichiometric formulation and should scale proportionally with the stoichiometric ratio. Second, because diffusivities of thiol and allyl monomers vary with molecular size and because the allyl monomers potentially homopolymerize, the depletion–replenishment effect is expected to occur even in stoichiometric formulations, albeit to a much lower degree.

The latter hypothesis is experimentally observable in Figure 1a, for which the monomer diffusivity ratio in the unpolymerized stoichiometric thiol–ene was estimated using the Stoke–Einstein equation to 1:1.13 of thiol:ene, where a weak variation of the concentration profile is discerned near the PFB. Moreover, because matching diffusivities of thiol and enes are expected to be very rare, it is probable that such concentration profiles are to be found in most photostructured thiol–ene copolymers.

A consequence of the above hypotheses is that reduction of the photomask feature dimensions is expected to cause increased monomer depletion in the non-illuminated region because downscaling leads to increased polymer structure surface to volume ratios (Figure 3c).

From the findings above, several additional consequences are expected. First, because a denser cross-linking leads to an increased refractive index^14^, a refractive index gradient automatically forms across the illumination interface. The qualitative model in Figure 4 suggests that such interfaces would be diffuse in stoichiometric thiol–ene formulations but sharp in off-stoichiometric formulations. This effect is promising for holographic data recording or photonic applications, for which high refractive contrast is desired^19,20^. Second, the formation of a highly cross-linked wall in zone C and the depletion of deficient groups in zone B improve the image transfer from mask to polymer during structuring by preventing broadening (Figure 2 and Supplementary Figure S3), which is useful for photostructuring of high aspect ratio and dense microstructures^21^.

Finally, the mechanism also suggests that structure downscaling leads to a global depletion of the deficient ene groups in the non-illuminated region that ultimately could prevent gelation regardless of exposure time (Figure 3c). Consequently, high-resolution photostructuring of very small features should be possible. This effect needs to be verified using high-precision exposure equipment that minimizes stray light, which otherwise obscures the diffusion effect.

In summary, here we have explained and provided supportive experimental results for the occurrence of stoichiometry-induced effects in the photostructuring of OSTE polymers. The formation of cross-linking density and material property gradients was reported and explained. The results clearly indicate that the underlying replenishment and depletion mechanism offers promising perspectives for holographic data storage and photonic applications.
