# Supplementary material for: Off-stoichiometry improves the photostructuring of thiol–enes through diffusion-induced monomer depletion
Source: Microsyst Nanoeng. 2016 Feb 15;2:15043. doi: 10.1038/micronano.2015.43 (PMC6444721; doi:10.1038/micronano.2015.43)
Supplement: Supplementary Figures [file micronano201543-s1.pdf]

Supplementary file

Off-stoichiometry improves photostructuring of thiol-enes through diffusion-induced monomer depletion

PREPARATION OF SAMPLES

Figure S1 shows how the samples for Raman microscopy were prepared.

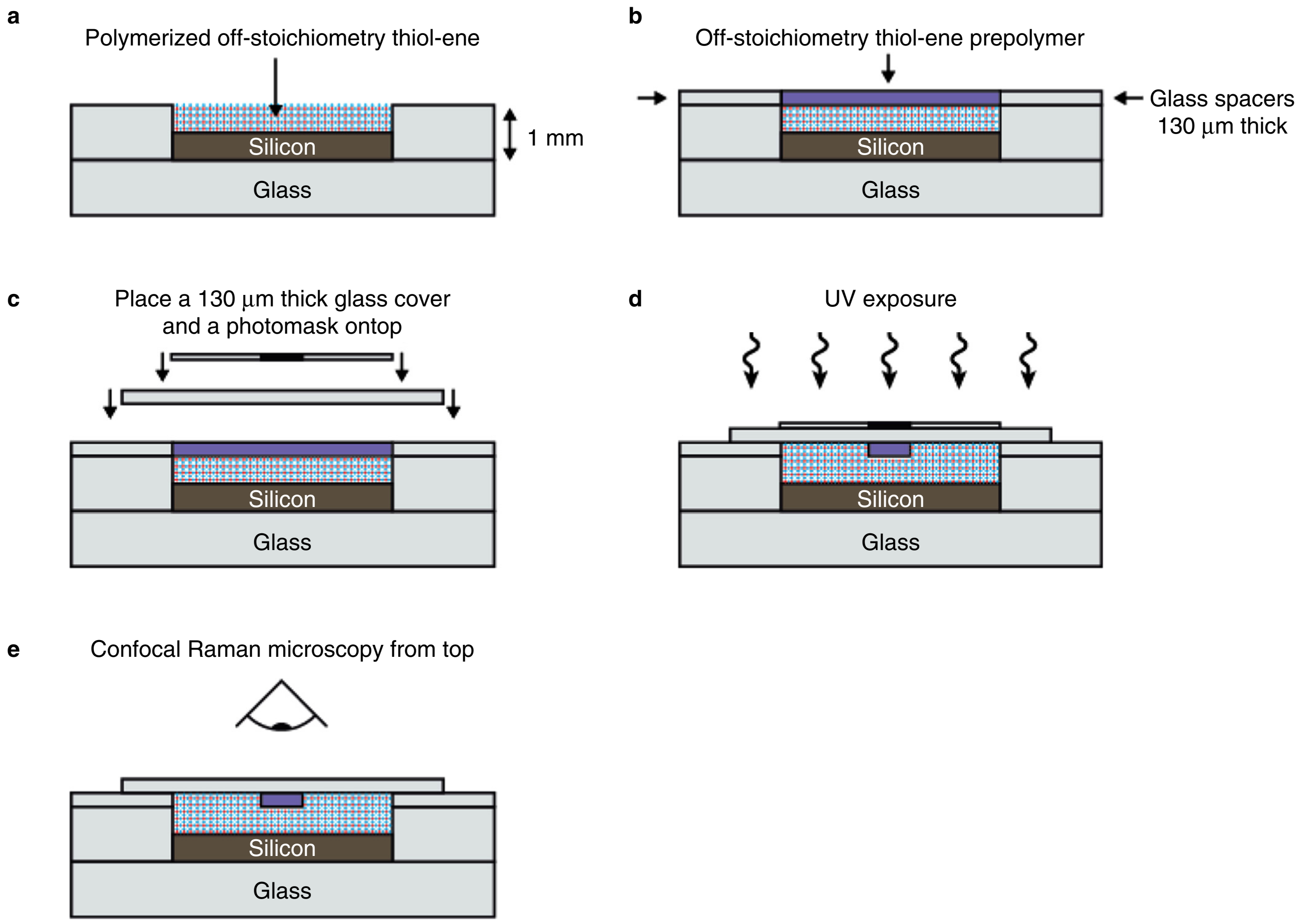

Figure S1 Preparation procedure for samples for confocal Raman microscopy. (**a**) Off-stoichiometry thiol-ene is polymerized onto a silicon substrate within a glass mold. (**b**) A 130 μm thick off-stoichiometry thiol-ene prepolymer layer is deposited onto the polymerized thiol-ene. The thickness is controlled by using glass spacers. (**c**) To enable a flat layer of prepolymer a glass cover is put on top. A photomask is placed on top of the cover glass. (**d**) UV exposure through the photomask and the glass cover enables photostructuring in the polymer layer. (**e**) Confocal Raman microscopy is performed by scanning through the top cover glass.

DEPENDENCE OF DEFICIENT MONOMER DEPLETION ON THE STRUCTURE VOLUME

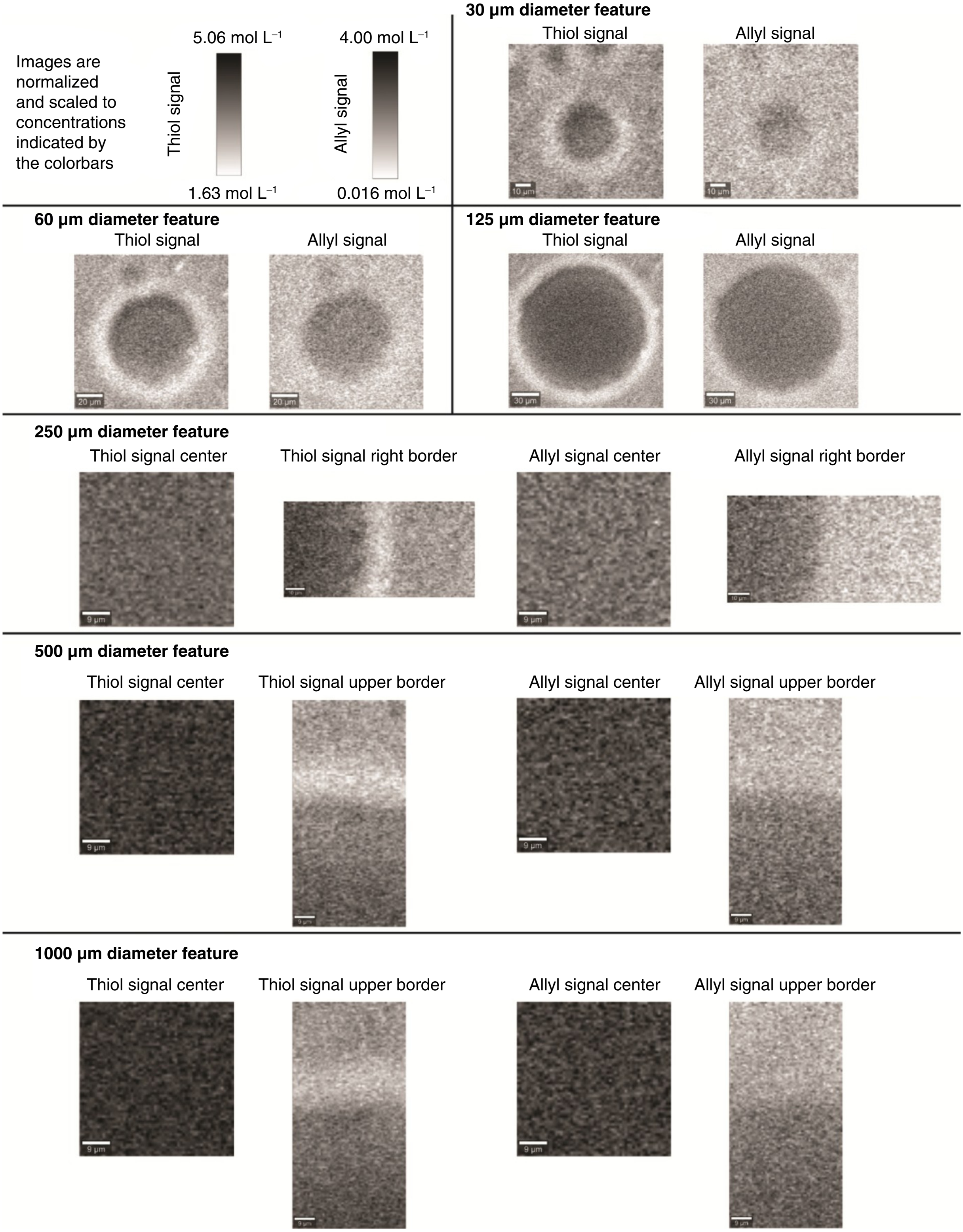

PHOTOSTRUCTURING QUALITY

The capability of off-stoichiometry thiol-ene formulations to prevent structure broadening was investigated by measuring the spatial shift of visible structure interfaces produced via photostructuring at different stoichiometries and exposure time (Figure S3, S4 and Table S1). For the image of the developed pillar structures (Figure S3), stoichiometric and 80% thiol excess off-stoichiometric polymers were photostructured with 20 s. UV exposure on a silicon wafer and developed using Butyl Acetate (Sigma-Aldrich Co., USA) under constant agitation for 2 min at room temperature, followed by rinsing using Isopropyl Alcohol and drying using nitrogen gas. The photopattern consisted of 50 µm diameter bright field circular patterns, i.e. the oppo-site polarity compared to the stoichiometry and depletion experiments.

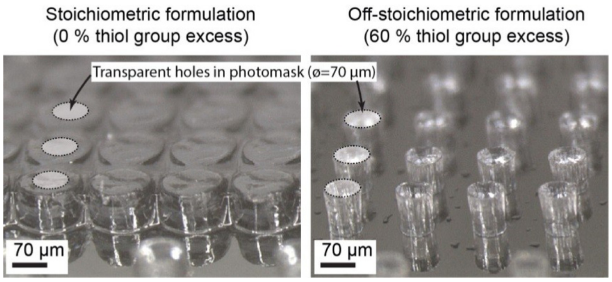

Figure S3 Photographs show photolithographically defined 70 µm-diameter pillars using stoichiometric (left) and off-stoichiometric (60 % thiol excess, right) thiol-ene formulations after 20 s UV expo-sure in a 150 µm-thick layer.

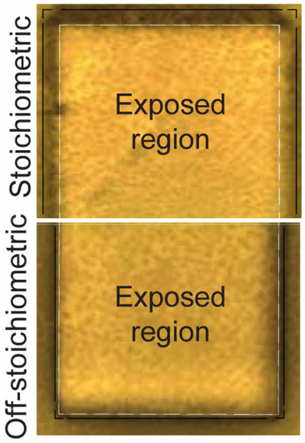

Figure S4 Microscope image of the pattern used for measurement of the broadening across infinite line patterns during photostruc-turing. The distance between the vertical interfaces was measured (and halved) to measure the broadening for each image. The location of the interface was found by plotting the signal profile for an image and set the interface location where the signal reached 50% of the signal in the exposed region using the software Image-J (National Institutes of Health, USA). Measurements were perfor-med on 6 samples per stoichiometry ratio for each of two sets of experiments.

Table S1 Mixing ratio for the levels of thiol group excess used for the broadening experiments.

Thiol group excess [%]	PETMA [%w/w]	TATATO [%w/w]
0	56.55	43.45
20	60.96	39.04
40	64.56	35.44
60	67.56	32.44
80	70.08	29.92

INVESTIGATION OF TEMPERATURE, LIGHT INTENSITY AND MASK EFFECTS

Using the confocal Raman microscope, vertical cross-sections (yz-section) were acquired across the photomask feature boundary (PFB) on three samples: two samples were prepared using an off-stoichiometric formulation with 80% thiol excess and illuminated while maintained at room temperature, 24°C, and at low temperature, 11°C, respectively; and one sample was prepared using a stoichiometric formulation and illuminated at room temperature, 24°C.

The resulting images are displayed in Figure S5a to f in the upper-left corner. On these images, the dashed white line shows the position of the PFB and the position of three cross-sections, described below. The image in the upper-right corner of Figure S5a and e are shown and discussed in the main article in Figure 1.

Compensation of depth-induced loss of Raman signal

For each image, a vertical cross-section (in green on the images) was recorded along a straight vertical line in the area of the sample where the polymer was not illuminated. Each of the data points in the cross-sections is the result of an average over a 10 pixels range centered over the cross-sectional line. The cross-sections are plotted for each image in green in the lower-left corner plot in Figure S5.

It can be seen that after rising to a maximum, the signal decreases exponentially with depth. The initial increase to the maximum represents the position of the top surface of the sample and the maximum represent the position at which point the laser voxel is entirely inside the sample. From that position on and when scanning deeper in the sample, the intensity of the Raman scattering decreases because of attenuation of the laser excitation and of the Raman scattering.

To alleviate for such signal loss and to be able to compare horizontal cross-sectional profiles at different depths, the loss of signal was compensated using the following procedure.

First, the maximum intensity point was located (using Matlab max() function) on the vertical cross-section described above (for the green profile). Then, a cross-section was extracted from the part of the profile ranging from the identified maximum to the bottom of the sample. The signal of the cross-section was then truncated from its first and last 10%, i.e. keeping the data between 10% and 90% of the depth from the maximum to the bottom. This data range was then fitted with an exponential curve, as shown in dashed black lines in Figure S5, over the cross-sectional plot, using the fit() function in Matlab.

Finally, the cross-sectional image data ranging from the maximum signal position to the bottom of the cross-section was divided by the value of the exponential fit in that range, effectively compensating for the depth-induced signal loss, as shown in the cross-sectional plot in magenta in Figure S5. The resulting image is shown in the low-right corner of Figure S5a to f.

Last, two horizontal cross-sections were recorded on both the original image and the depth-corrected image, at depths of 7.5 μm and 45 μm from the image top, respectively. These cross-section are plotted in the upper-right graphs in Figure S5, using blue for 7.5 μm deep profile on both images (since they are not affected by the depth correction), yellow for the 45 μm deep profile on the original image, and red on the depth-corrected image at the same depth of 45 μm. The location of these cross-section profiles are also shown on the respective images with the same color.

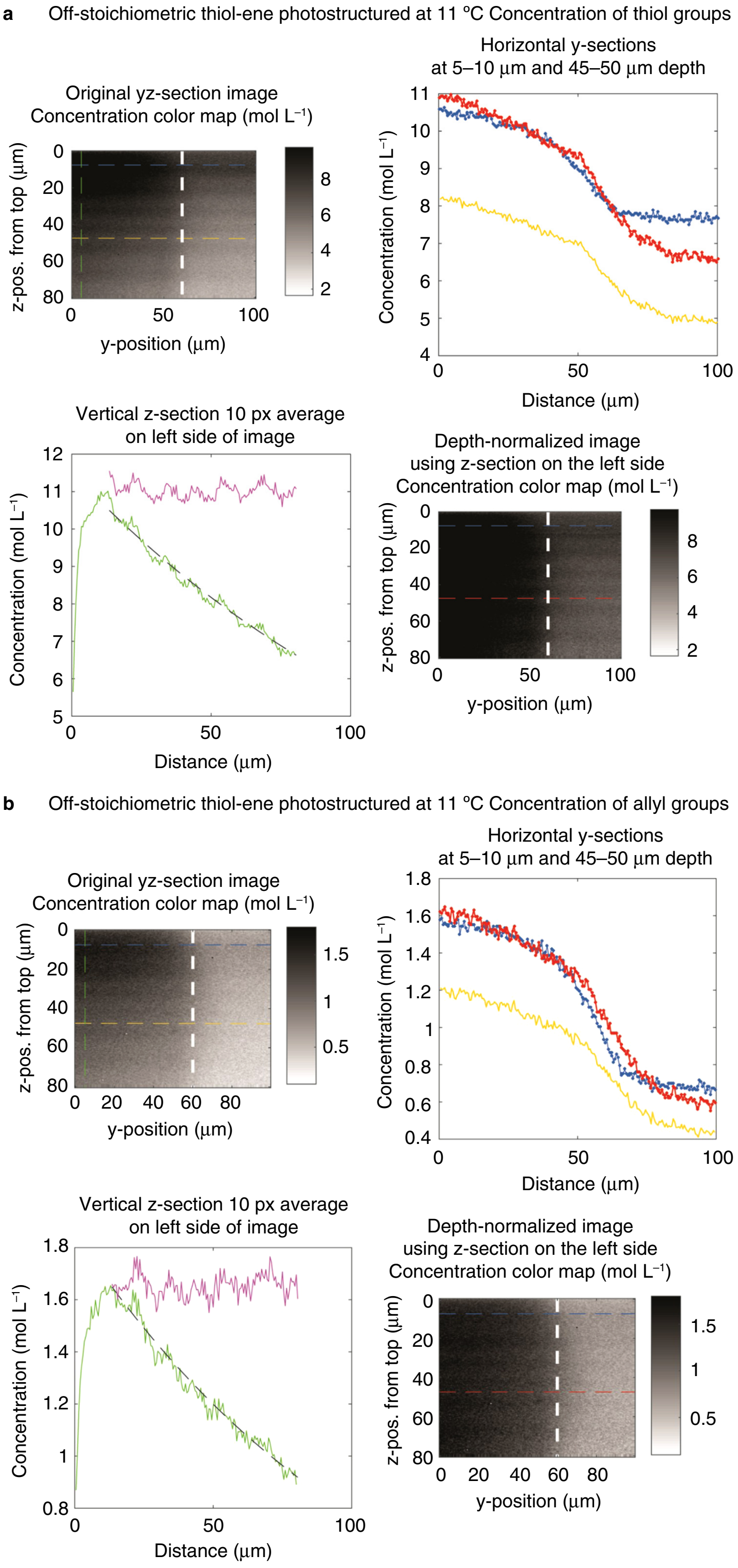

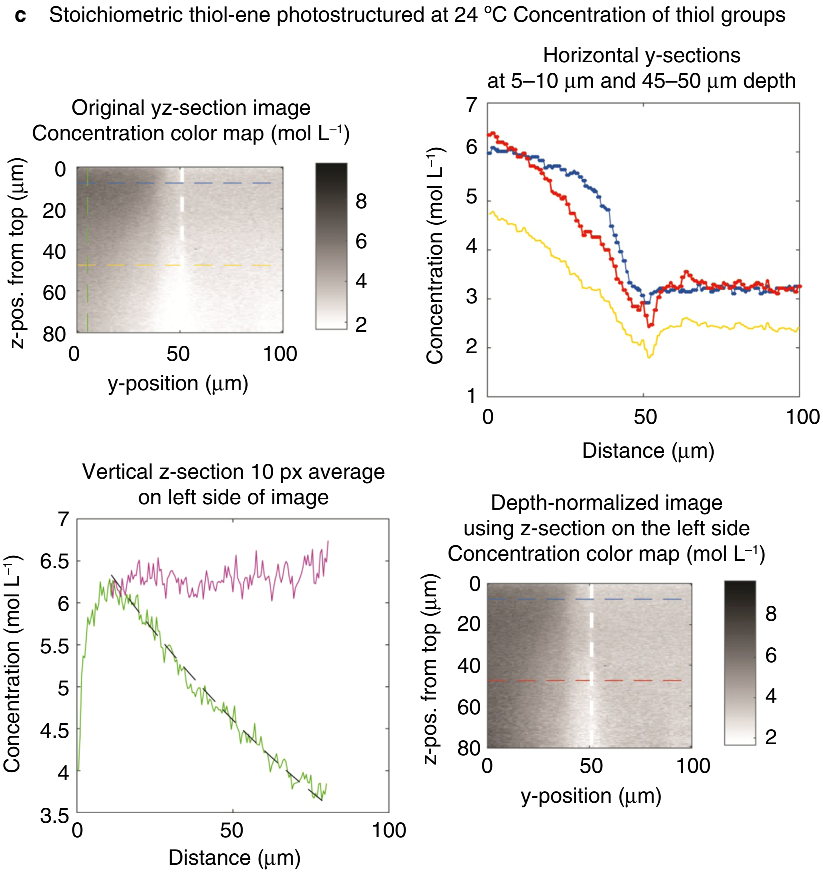
c Stoichiometric thiol-ene photostructured at 24 °C Concentration of thiol groups
Original yz-section image
Concentration color map (mol L⁻¹)
z-pos. from top (µm)
y-position (µm)
Horizontal y-sections
at 5–10 µm and 45–50 µm depth
Concentration (mol L⁻¹)
Distance (µm)
Vertical z-section 10 px average
on left side of image
Concentration (mol L⁻¹)
Distance (µm)
Depth-normalized image
using z-section on the left side
Concentration color map (mol L⁻¹)
z-pos. from top (µm)
y-position (µm)

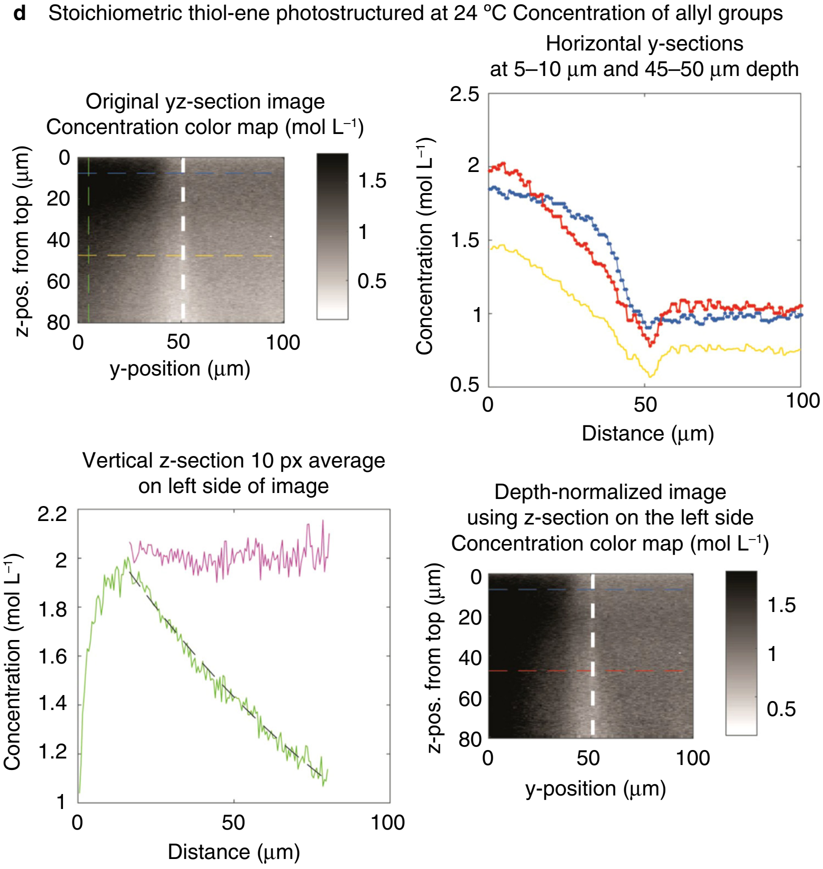
d Stoichiometric thiol-ene photostructured at 24 °C Concentration of allyl groups
Original yz-section image
Concentration color map (mol L⁻¹)
z-pos. from top (µm)
y-position (µm)
Horizontal y-sections
at 5–10 µm and 45–50 µm depth
Concentration (mol L⁻¹)
Distance (µm)
Vertical z-section 10 px average
on left side of image
Concentration (mol L⁻¹)
Distance (µm)
Depth-normalized image
using z-section on the left side
Concentration color map (mol L⁻¹)
z-pos. from top (µm)
y-position (µm)

e Off-stoichiometric thiol-ene photostructured at 24 °C Concentration of thiol groups

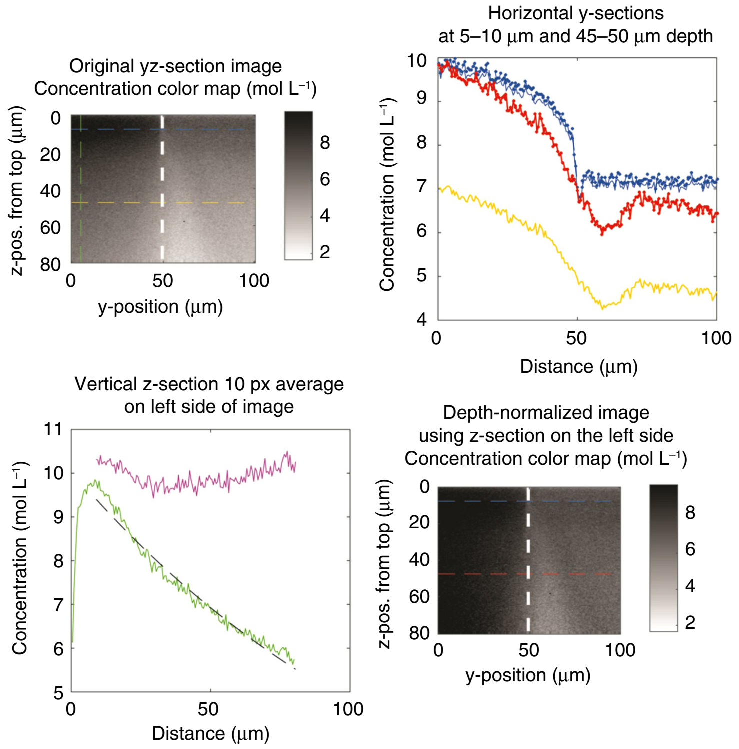

f Off-stoichiometric thiol-ene photostructured at 24 °C Concentration of allyl groups

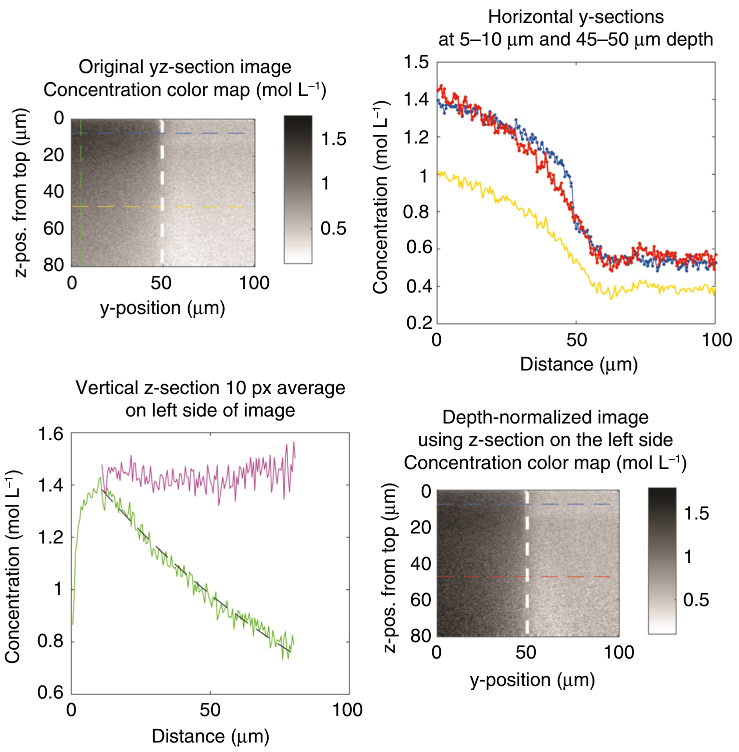

Figure S5(a)–(f) Showing output from an image processing script used on Raman microscopy images. The upper left image shows the original Raman yz-scan across the PFB of the sample; the upper right image shows the resulting graphs from the different horizontal cross-sections recorded from both the original (unprocessed image) and the vertical intensity corrected image (regions of interest are indicated as dashed lines); the lower left graph indicates how a curve was fitted to the vertical intensity decay and how this fitted curve was used for correcting the intensity of the original image; lower right image shows the resulting yz-scan after image vertical intensity correction. The white vertical dashed line indicates the PFB and the non-illuminated region is to the left of the PFB line. The image greyscale is mapped to functional group concentration (as indicated by the scale bar).

CONFOCAL RAMAN MICROSCOPY

The Raman spectra of both monomers were measured in order to create a filter to measure intensity from only thiol (PETMA) and allyl (TATATO) peaks, as displayed in Figure S6. The thiol peak was identified at ~2575 cm^{-1} and the Raman scattering signal was integrated between 2507 cm^{-1} and 2647 cm^{-1}. The allyl peak was identified at ~1650 cm^{-1} and the Raman scattering signal was integrated between 1627 cm^{-1} and 1667 cm^{-1}. The carbon-oxygen double bond peak was identified at ~1750 cm^{-1}. Since such inert C=O chemical bonds are present on both the thiol and allyl monomers, this peak was used for normalization of the spectra (for more information on how this peak was used for normalization see Figure S7).

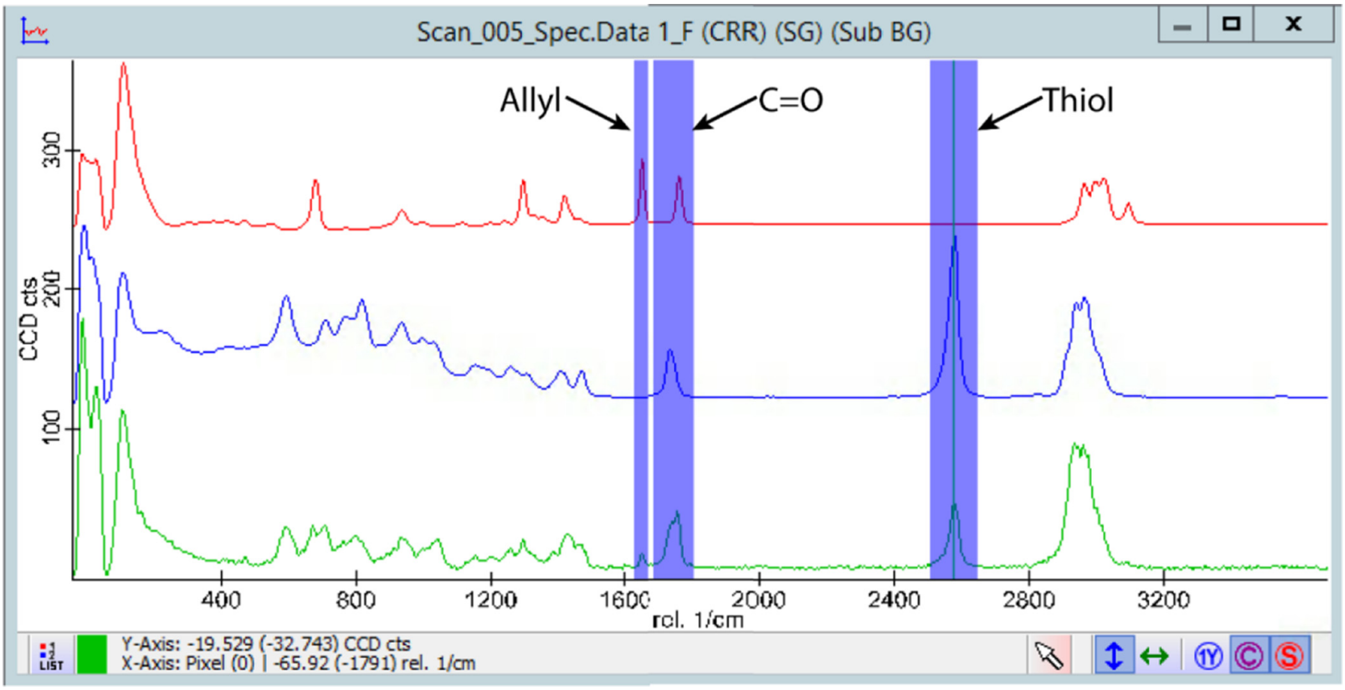

Figure S6 Raman spectrum for both monomers (PETMA and TATATO), and the integration region for the thiol, allyl groups, and C=O groups.

DATA PROCESSING: NORMALIZATION AND SCALING

Processing of spectra

After acquisition, the spectra were processed, using the built-in tools in the Raman software "Project Four" from WiTec, with a cosmic ray removal (CRR) filter (parameters used were: filter size: 2 px and dynamic factor: 8) and with a Savitzki-Golay smoothening filter (parameters used were: left: 5 px, right: 5 px, order: 5 and derivative: 0). Thereafter, the background was removed using polynomial of order 6 fitted to specific portions of the spectra, representative of the background signal and defined with a mask filter, in the range: -48 to -39, 54 to 62, 630 to 650, 1543 to 1589, 1937 to 2399 and 3209 to 3691 rel. cm^{-1}, as shown in Figure S7a. The background removal procedure yielded good and reproducible results for each measured spectra, especially in the spectral range of the peaks of interest, from 1600 rel. cm^{-1} to 2700 rel. cm^{-1} (Figure S7b).

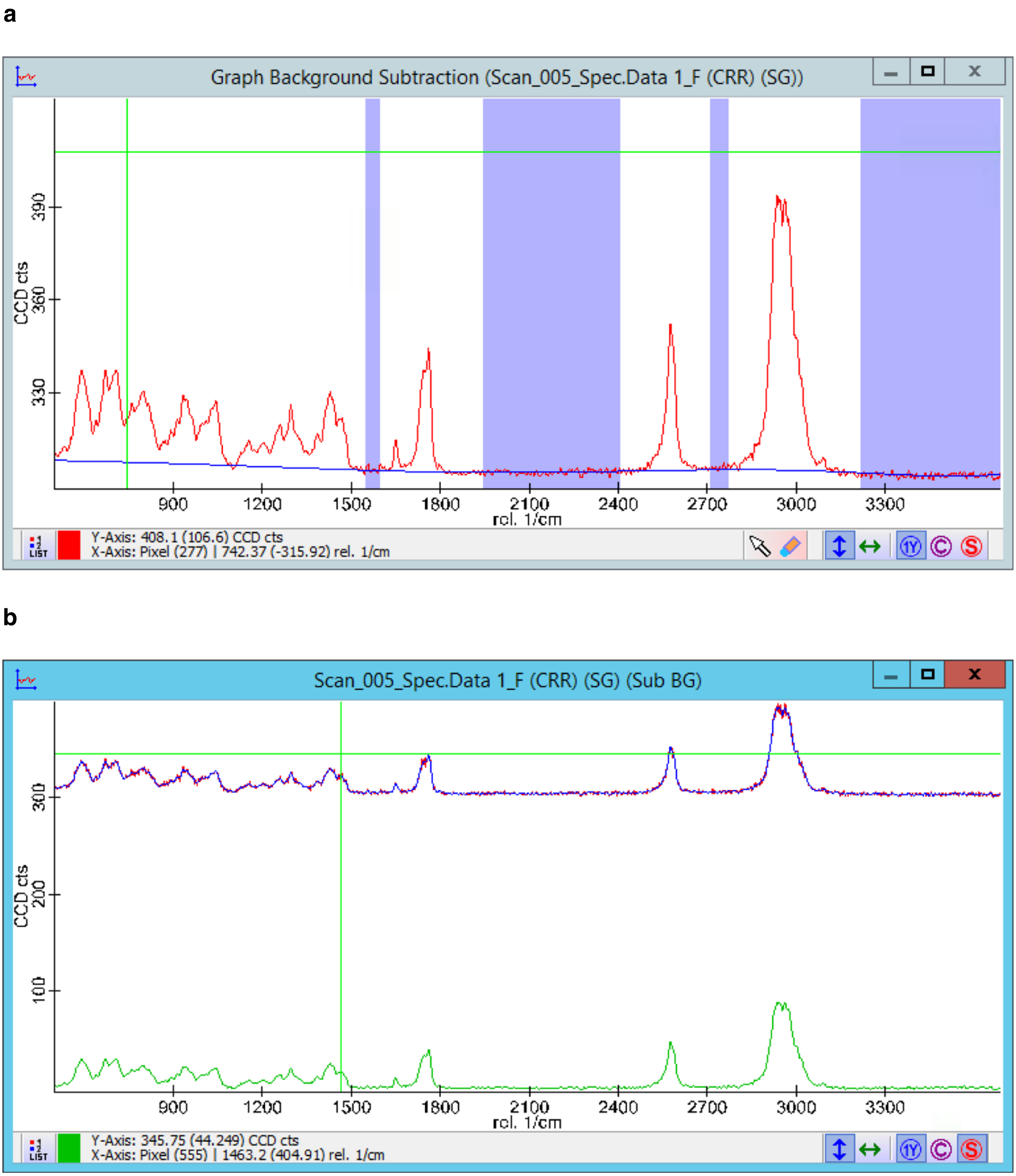

Figure S7 (**a**) Example of background removal: the original spectra is red, the fitting mask is shown as blue area, and the fitted background polynomial in blue. (**b**) Example of spectra before processing, in red; after cosmic ray removal and Savitzki-Golay filtering, in blue; and after background removal in green.

Normalization of spectra

Each spectrum was then normalized to make them comparable by alleviating for variation of the laser excitation intensity and for signal attenuation induced by the scan depth and materials.

For this, we looked for a spectral peak that would remain unaltered during polymerization, which could serve as reference for normalizing the spectra.

Since the C=0 bond are present on both monomers, three bond on TATATO and four bond on PETMA, and are inert during polymerization, the Raman signal for this group is expected to remain the same in the uncured and cured state of the polymer, assuming negligible signal variations due to the changing polymer matrix. The C=O peak was identified around 1750 rel. cm^{-1} in the spectra. However, it was observed that the peak induced by the C=O bond carried by the PETMA is slightly shifted compared to the peak from the C=O bond carried by the TATATO monomers, as seen on Figure S7c.

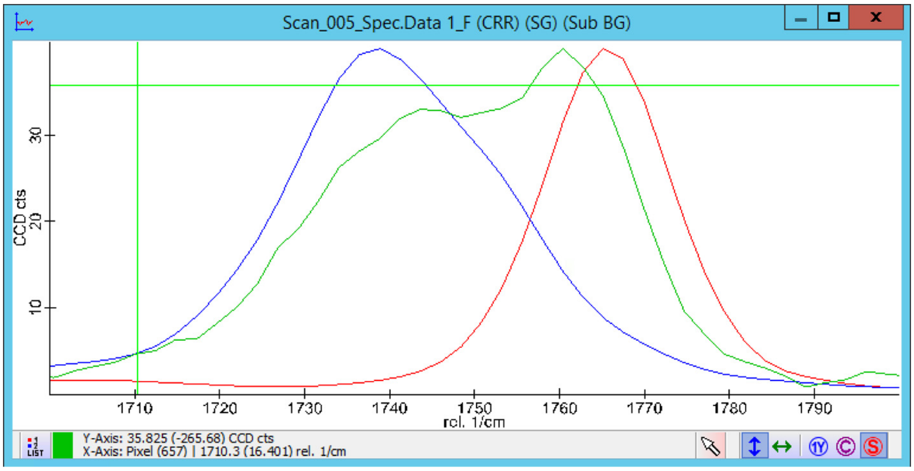

Figure S7c Shift of spectral peak for the C=O chemical bond for measurement on pure TATAO, pure PETMA and a sample made of off-stoichiometry thiol-ene with 80% molar excess of thiol groups compared to allyl groups. Note, the vertical scale correspond to the green spectra, the other spectra are scale for better visualization of the peak shift.

Therefore, the peak was fitted with two Gaussian curves to perform a deconvolution of the peak corresponding to the C=O on PETMA monomers and to those on TATATO monomers. The fitting was performed using the built-in fitting tool in the Raman software "Project Four" from WiTec.

The fitting parameters were: Tolerance: 10^8; Max # of iteration: 200; Number of function: 2; Extract peak width kind: Standard deviation; Extract intensity kind: Area; Extract sort kind: None. A fitting mask was used to restrict the fitting to the range 1680 to 1920 rel. cm^{-1}. The curve model used was a double Gauss curve, each based on the following function:

$$y = y_0 + \Sigma_{0\to 1}^{i} I_i \cdot \exp\left[-\tfrac{1}{2}\left(\frac{(x - x_0(i))}{\sigma(i)}\right)^2\right]$$

Start values where provided to the software to enable a reproducible fitting on all spectra. The y_o value was force to 0, and the following values was provided for each Gaussian:

Table S2

Start values	Gaussian 1	Gaussian 2
x_o	1740	1760
s	16	8
I_i	500	300

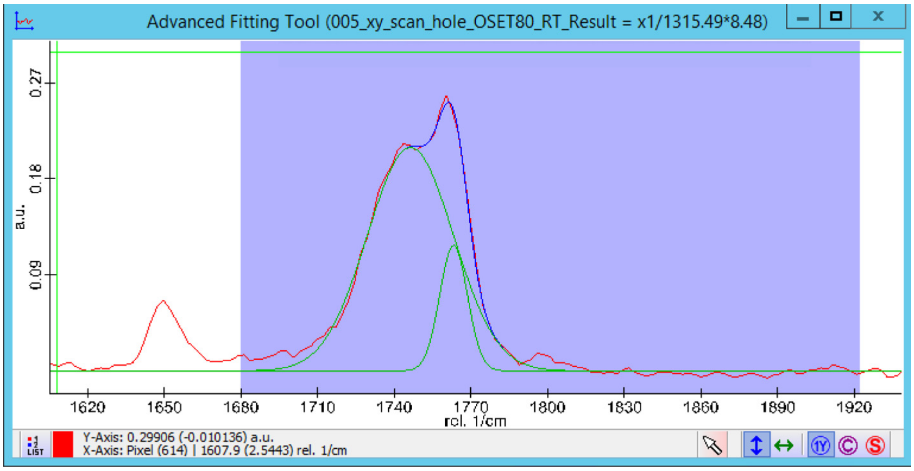

Figure S7d Example of the fitting of the C=O peak with two Gaussian curves for an off-stoichiometric sample with 80% thiol excess.

An example of the fitting results is shown in Figure S7d.

The area under the curve fitting for the C=O bond on the PETMA monomers, $A_{C=0\text{-Thiol mon.}, i}$, – the green fitting curve visible on the left in the C=O peak in Figure S7d, was used to normalize each the spectra, $S_{0,\ i}$. This peak was preferred to the smaller C=0 peak from the TATATO, shifted to the right. The reason behind the peak size difference lies in the number of bonds per monomer and to the stoichiometry of the formulation.

In the case of image spectra, the normalization peak area was performed on an averaged subset of the image spectra, chosen in a uniform area of the image, for instance the non-polymerized area, to ensure that refractive index or density change would not introduce any bias.

Since the concentration of these groups vary between the formulations, the normalized spectra was also multiplied by the molar concentration of the thiol monomer carried C=O bond in that particular formulation, to obtain comparable spectra between formulations.

The final normalized spectrum is therefore calculated as:

$$S_{\text{norm.},i} = \frac{c_{C=O\text{ on Thiol mon.},i}}{A_{\text{C=0 on Thiol mon.,i}}} \cdot S_{0,i}$$

Image extraction from spectra

Images, $IMG_{k,i}$ – with k standing for Allyl or Thiol –, showing the spatial distribution for the Allyl and Thiol peaks were then extracted using sum filtering on each spectra, with two sum filters in the range 1627 – 1667 rel. cm^{-1} and 2507 2647 rel. cm^{-1}, for the respective Allyl and Thiol peaks.

Scaling of intensity to molar concentration

In an attempt to make our measurement quantitative, the intensity in the images was then scaled to the molar concentration of the respective thiol and allyl groups in the formulations used for each sample, by dividing the intensity by the slope, m_k, of the linearization of the Raman signal for the respective peaks.

Raman signal linearization was performed using linear regression on point spectra measurements of samples, each prepared using either of the non-polymerized pure monomers or non-polymerized and photoinitiator free stoichiometric and off-stoichiometric formulations. On these samples, the thiol and allyl spectral intensities were obtained using the sum filter described above. Five point-spectra per sample were taken and averaged and the obtained values are plotted against the respective molar concentrations in Figure S7e to f.

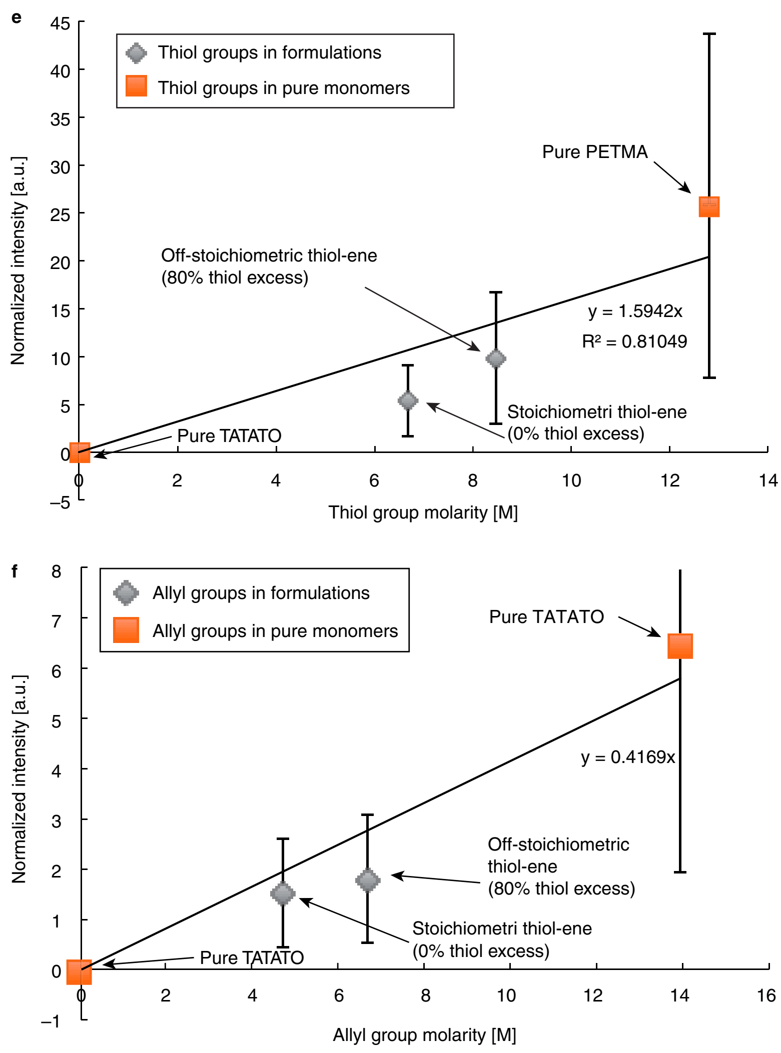

Figure S7e-f Linearization of the (**e**) thiol and (**f**) allyl Raman peak intensity sum to molar concentration.

The slopes was obtained from the linear regression:

$$m_{\text{allyl}} = 0.417 \pm 11\% \qquad ()$$

$$m_{\text{thiol}} = 1.594 \pm 18\% \qquad ()$$

The relatively large error is due to the normalization of the spectra, in which the fitting of the C=O bond peak induces a large error, on average > 30%, despite very good correlation value, on average > 0.99.

The obtained slopes were used to scale each image, $IMG_{k,i}$, according to:

$$IMG_{k,i,\text{scaled}} = m_{k,i} \cdot IMG_{k,i}, \qquad ()$$

Image cross-sections

Cross-sections were extracted using the built-in cross-section tool, using a straight line drawn across the photomask feature boundary (PFB). To get less noise sensitive data, the cross-section is the results of averaging over a specific width centered on the cross-section line. For straight interfaces, the average was taken on a 20 µm wide range perpendicular to the cross-section line. In the case of circular patterns down to 50 µm diameter, the range width was limited to 10 µm; and for the pattern with a 30 µm diameter, to 5 µm.

Influence of illumination duration on the thiol and allyl Raman measurements

The influence of the illumination duration, i.e. exposure time, was observed be point measurements on two polymerized sample, prepared with stoichiometric and off-stoichiometric thiol-ene formulations. The average of five point-spectra measurements are shown in Figure S7g to h.

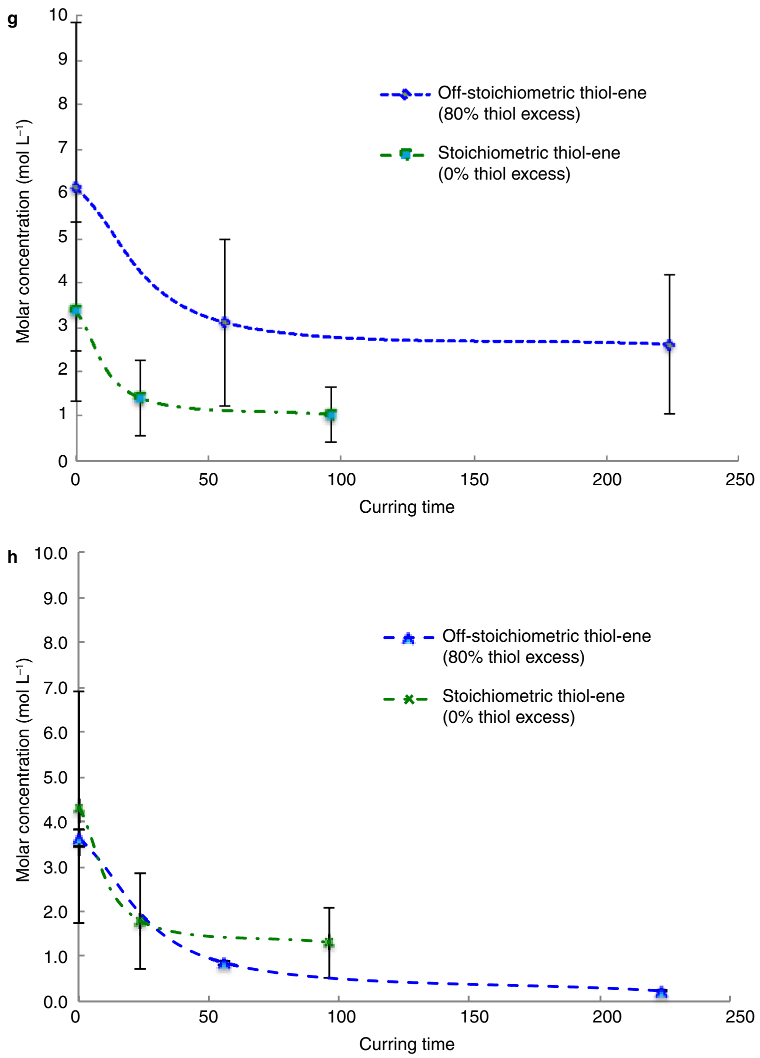

Figure S7g-h Molar concentrations of (**g**) thiol and (**h**) allyl as a function of the illumination duration for two samples prepare using stoichiometric and off-stoichiometric thiol-ene formulations, respectively. Lines are for visual guidance only.

Figure S7i demonstrates that the scaled and normalized spectra measurements are able to capture the consumption of the functional allyl and thiol groups during polymerization. It also shows that complete curing of the monomer only occur with extended time, beyond those used for the measurements. It is important to note that the reaction rate is very dependent on the amount of photoinitiator and on the stoichiometry.

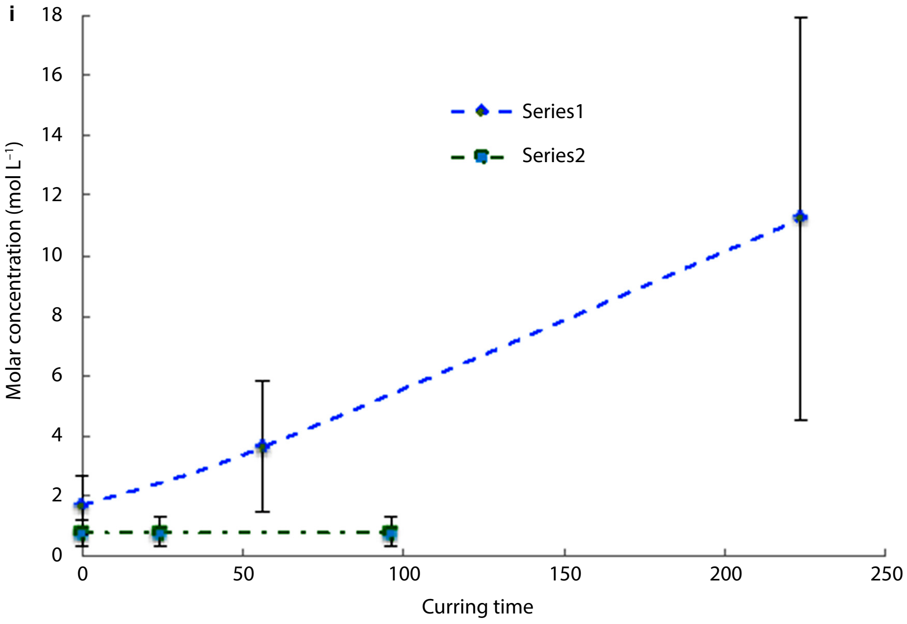

Figure S7i Ratio of the thiol over allyl molar concentrations, plotted in Figure S7 (**g**) to (**h**), as a function of the illumination duration.

The ratio of the values shown in Figure S7g to h are plotted in Figure S7i. This shows that a constant ratio of 1 is maintained during polymerization of the stoichiometric thiol-ene sample, while the ratio diverges towards infinity for the off-stoichiometric thiol-ene sample, as the excess of thiol remains while the deficient allyl groups are gradually all consumed.
